# 1,1′-Binaphthyl-2,2′-dicarboxylic acid–urea (1/1)

**DOI:** 10.1107/S1600536808028997

**Published:** 2008-09-17

**Authors:** Lidiya Izotova, Jamshid Ashurov, Samat Talipov, Bakhtiyar Ibragimov, Edwin Weber

**Affiliations:** a Institute of Bioorganic Chemistry, Academy of Sciences of Uzbekistan, H. Abdullaev Street 83, Tashkent 100125, Uzbekistan; b Institute für Organische Chemie, TU Bergakademie Freiberg, Leipziger Strasse 29, D-09596 Freiberg/Sachsen, Germany

## Abstract

In the title co-crystal, C_22_H_14_O_4_·CH_4_N_2_O, the 1,1′-binaphthyl-2,2′-dicarboxylic acid (BNDA) and urea mol­ecules are connected *via* a system of hydrogen bonds into a chiral two-dimensional polymeric structure parallel to the (001) plane. As the crystal is centrosymmetric, it consists of alternately stacked BNDA–urea layers of opposite chirality. The urea H atoms *trans* to the C=O group are bonded in a chelating mode [*R* ^1^_2_(6)] to the carbonyl O atom from one of the carboxylic acid groups which, in turn, acts as the donor of an O—H⋯O hydrogen bond to another urea mol­ecule. The [010] chains thus formed are further connected *via* an *R* ^2^_2_(8) hydrogen-bond motif formed between urea and the second carboxylic acid group of BNDA.

## Related literature

For information on inclusion compounds of 1,1′-binaphthyl-2,2′-dicarboxylic acid, see: Weber (1996 ▶). For the synthesis of 1,1′-binaphthyl-2,2′-dicarboxylic acid, see: Weber *et al.* (1984 ▶).
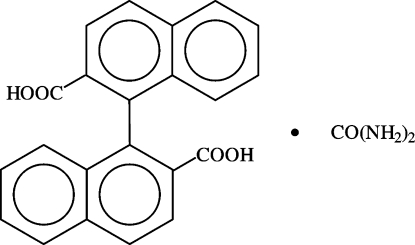


## Experimental

### 

#### Crystal data

C_22_H_14_O_4_·CH_4_N_2_O
*M* *_r_* = 402.39
Monoclinic, 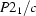

*a* = 9.2560 (19) Å
*b* = 12.033 (2) Å
*c* = 17.958 (4) Å
β = 102.40 (3)°
*V* = 1953.5 (7) Å^3^
*Z* = 4
Mo *K*α radiation
μ = 0.10 mm^−1^
*T* = 293 (2) K
0.21 × 0.17 × 0.12 mm

#### Data collection

Stoe STADI4 diffractometer
Absorption correction: none
3416 measured reflections
3416 independent reflections
2874 reflections with *I* > 2σ(*I*)
3 standard reflections every 100 reflections intensity decay: 2.1%

#### Refinement

*R*[*F* ^2^ > 2σ(*F* ^2^)] = 0.044
*wR*(*F* ^2^) = 0.117
*S* = 1.09
3416 reflections
296 parameters
H atoms treated by a mixture of independent and constrained refinement
Δρ_max_ = 0.21 e Å^−3^
Δρ_min_ = −0.18 e Å^−3^

### 

Data collection: *STADI4* (Stoe & Cie, 1997 ▶); cell refinement: *STADI4*; data reduction: *X-RED* (Stoe & Cie, 1997 ▶); program(s) used to solve structure: *SHELXS97* (Sheldrick, 2008 ▶); program(s) used to refine structure: *SHELXL97* (Sheldrick, 2008 ▶); molecular graphics: *XP* (Siemens, 1994 ▶); software used to prepare material for publication: *SHELXL97*.

## Supplementary Material

Crystal structure: contains datablocks I, global. DOI: 10.1107/S1600536808028997/gk2167sup1.cif

Structure factors: contains datablocks I. DOI: 10.1107/S1600536808028997/gk2167Isup2.hkl

Additional supplementary materials:  crystallographic information; 3D view; checkCIF report

## Figures and Tables

**Table 1:** Hydrogen-bond geometry (Å, °)

*D*—H⋯*A*	*D*—H	H⋯*A*	*D*⋯*A*	*D*—H⋯*A*
N1—H1*N*⋯O4	0.89 (3)	2.25 (3)	3.046 (3)	149 (2)
N1—H2*N*⋯O1^i^	0.96 (3)	2.10 (3)	3.048 (3)	166 (2)
O2—H2⋯O5^ii^	1.00 (3)	1.63 (3)	2.606 (2)	162 (2)
O3—H3⋯O5^iii^	0.98 (3)	1.70 (3)	2.637 (2)	160 (3)
N2—H3*N*⋯O4	0.88 (3)	2.17 (3)	3.001 (3)	157 (3)

Symmetry codes: (i) 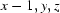; (ii) 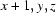; (iii) 
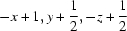
.

**Fig. 1.**
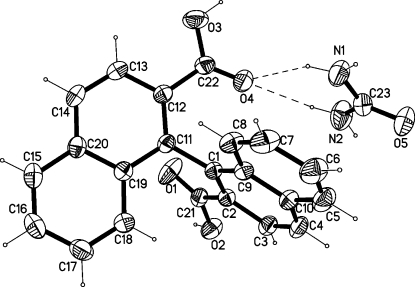
Perspective view of the asymmetric unit showing 40% probability displacement ellipsoids for the non-H atoms. Dashed lines represent hydrogen bonds.

**Fig. 2.**
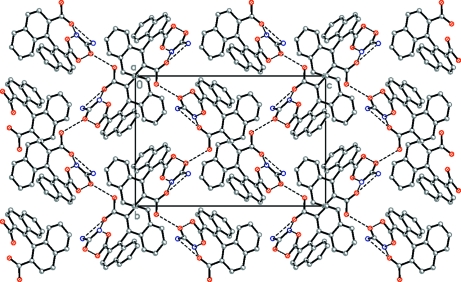
BNDA–urea hydrogen-bond assembly viewed down the a axis. H atoms have been ommited for clarity. Hydrogen bonds are shown as dashed lines. Red: oxygen, blue: nitrogen.

## supplementary crystallographic information

### Comment

Crystal engineering involves design and synthesis of solid-state structures with desired properties, based on an understanding and exploitation of intermolecular interactions. 1,1'-Binaphthyl-2,2'-dicarboxylic acid (BNDA) (Weber, 1996) and urea are well known supramolecular substrates forming a large variety of multicomponent crystals. To study supramolecular interactions in the BNDA–urea system co-crystals were prepared and their structure determined by X-ray structure analysis. The two compounds co-crystallize in a 1:1 molar ratio with molecules located in general position (Fig. 1). The BNDA adopts its usual conformation with the two naphthyl units nearly perpendicular [dihedral angle of 79.77 (5)°].

In the crystal structure, the urea molecule is involved in five hydrogen bonds - twice as an acceptor and three times as a donor (Table 1). One of the cis hydrogen atoms does not take part in conventional hydrogen bonding but is involved in a weak interaction with the π system of one of the naphthalene units. The carboxylic group O1,C21,O2 is bonded to the urea molecule via the cyclic *R*^2^_2_(8) motif. The other BNDA carboxylic group acts also as a donor to the urea carbonyl, however its O4 atom accepts two N—H···O hydrogen bonds which are a part of the *R*^1^_2_(6) motif (Fig. 2). The hydrogen bonds between urea and BNDA assemble molecules into layers parallel to (001). The hydrogen-bonded BNDA–urea layers are chiral and consist of homochiral BNDA molecules. However, the crystal is centrosymmetric and thus it is built from alternate stacks of layers of opposite chirality.

### Experimental

1,1'-Binaphthyl-2,2'-dicarboxylic acid was synthesized according to Weber *et al.* (1984). Slow evaporation of acetone/water solution of equimolar mixture of BNDA and urea resulted in colourless crystals stable in air and suitable for X-ray analysis.

### Refinement

H atoms from the OH and NH2 groups were located from difference Fourier maps and fully refined. The remaining H atoms were positioned geometrically (C—H 0.93 Å) and refined as riding on their carrier atoms with *U*_iso_(H) = 1.2*U*_eq_(C).

### Crystal data

**Table:**

C_22_H_14_O_4_·CH_4_N_2_O	*F*(000) = 840
*M**_r_* = 402.39	*D*_x_ = 1.368 Mg m^−^^3^
Monoclinic, *P*2_1_/*c*	Mo *K*α radiation, λ = 0.71073 Å
Hall symbol: -P 2ybc	Cell parameters from 25 reflections
*a* = 9.2560 (19) Å	θ = 10–25°
*b* = 12.033 (2) Å	µ = 0.10 mm^−^^1^
*c* = 17.958 (4) Å	*T* = 293 K
β = 102.40 (3)°	Prism, colourless
*V* = 1953.5 (7) Å^3^	0.21 × 0.17 × 0.12 mm
*Z* = 4	

### Data collection

**Table:**

Stoe STADI4 diffractometer	*R*_int_ = 0.000
Radiation source: fine-focus sealed tube	θ_max_ = 25.0°, θ_min_ = 2.8°
graphite	*h* = −11→10
ω/2θ scans	*k* = 0→14
3416 measured reflections	*l* = 0→21
3416 independent reflections	3 standard reflections every 100 reflections
2874 reflections with *I* > 2σ(*I*)	intensity decay: 2.1%

### Refinement

**Table:**

Refinement on *F*^2^	Secondary atom site location: difference Fourier map
Least-squares matrix: full	Hydrogen site location: inferred from neighbouring sites
*R*[*F*^2^ > 2σ(*F*^2^)] = 0.044	H atoms treated by a mixture of independent and constrained refinement
*wR*(*F*^2^) = 0.117	*w* = 1/[σ^2^(*F*_o_^2^) + (0.050*P*)^2^ + 0.871*P*] where *P* = (*F*_o_^2^ + 2*F*_c_^2^)/3
*S* = 1.09	(Δ/σ)_max_ < 0.001
3416 reflections	Δρ_max_ = 0.21 e Å^−^^3^
296 parameters	Δρ_min_ = −0.18 e Å^−^^3^
0 restraints	Extinction correction: SHELXL97 (Sheldrick, 2008), Fc^*^=kFc[1+0.001xFc^2^λ^3^/sin(2θ)]^-1/4^
Primary atom site location: structure-invariant direct methods	Extinction coefficient: 0.0083 (11)

### Special details

**Table:**

Geometry. All e.s.d.'s (except the e.s.d. in the dihedral angle between two l.s. planes) are estimated using the full covariance matrix. The cell e.s.d.'s are taken into account individually in the estimation of e.s.d.'s in distances, angles and torsion angles; correlations between e.s.d.'s in cell parameters are only used when they are defined by crystal symmetry. An approximate (isotropic) treatment of cell e.s.d.'s is used for estimating e.s.d.'s involving l.s. planes.
Refinement. Refinement of *F*^2^ against ALL reflections. The weighted *R*-factor *wR* and goodness of fit *S* are based on *F*^2^, conventional *R*-factors *R* are based on *F*, with *F* set to zero for negative *F*^2^. The threshold expression of *F*^2^ > σ(*F*^2^) is used only for calculating *R*-factors(gt) *etc*. and is not relevant to the choice of reflections for refinement. *R*-factors based on *F*^2^ are statistically about twice as large as those based on *F*, and *R*- factors based on ALL data will be even larger.

### Fractional atomic coordinates and isotropic or equivalent isotropic displacement parameters (Å^2^)

**Table:**

	*x*	*y*	*z*	*U*_iso_*/*U*_eq_	
O1	1.04973 (17)	0.78156 (13)	0.14183 (10)	0.0668 (5)	
O2	1.08667 (16)	0.61883 (12)	0.19956 (9)	0.0529 (4)	
O3	0.6667 (2)	1.06357 (13)	0.10984 (10)	0.0687 (5)	
O4	0.6785 (2)	0.89894 (13)	0.16344 (10)	0.0718 (5)	
C1	0.74291 (19)	0.71568 (14)	0.08151 (10)	0.0335 (4)	
C2	0.84438 (19)	0.65851 (14)	0.13592 (10)	0.0351 (4)	
C3	0.7992 (2)	0.56422 (15)	0.17243 (11)	0.0407 (4)	
H3A	0.8687	0.5252	0.2080	0.049*	
C4	0.6563 (2)	0.52994 (16)	0.15642 (11)	0.0450 (5)	
H4A	0.6288	0.4687	0.1818	0.054*	
C5	0.3993 (2)	0.55259 (17)	0.08479 (13)	0.0531 (5)	
H5A	0.3696	0.4931	0.1109	0.064*	
C6	0.2983 (2)	0.60591 (18)	0.03102 (15)	0.0587 (6)	
H6A	0.1999	0.5835	0.0211	0.070*	
C7	0.3414 (2)	0.69477 (17)	−0.00973 (14)	0.0544 (6)	
H7A	0.2720	0.7297	−0.0476	0.065*	
C8	0.4844 (2)	0.73021 (16)	0.00577 (12)	0.0448 (5)	
H8A	0.5112	0.7896	−0.0215	0.054*	
C9	0.5929 (2)	0.67850 (14)	0.06262 (10)	0.0364 (4)	
C10	0.5492 (2)	0.58588 (15)	0.10188 (11)	0.0403 (4)	
C11	0.78527 (18)	0.80838 (14)	0.03449 (10)	0.0333 (4)	
C12	0.75590 (19)	0.91925 (15)	0.04525 (10)	0.0364 (4)	
C13	0.7863 (2)	1.00037 (15)	−0.00665 (11)	0.0439 (5)	
H13A	0.7633	1.0744	0.0001	0.053*	
C14	0.8480 (2)	0.97206 (16)	−0.06573 (12)	0.0465 (5)	
H14A	0.8681	1.0270	−0.0985	0.056*	
C15	0.9495 (2)	0.82851 (19)	−0.13856 (11)	0.0491 (5)	
H15A	0.9765	0.8828	−0.1698	0.059*	
C16	0.9756 (2)	0.7203 (2)	−0.15173 (12)	0.0546 (6)	
H16A	1.0196	0.7010	−0.1918	0.065*	
C17	0.9363 (2)	0.63771 (19)	−0.10509 (12)	0.0521 (5)	
H17A	0.9518	0.5634	−0.1152	0.062*	
C18	0.8756 (2)	0.66547 (16)	−0.04486 (11)	0.0423 (5)	
H18A	0.8524	0.6096	−0.0136	0.051*	
C19	0.84714 (18)	0.77740 (15)	−0.02885 (10)	0.0342 (4)	
C20	0.88207 (19)	0.86064 (16)	−0.07834 (10)	0.0381 (4)	
C21	1.0017 (2)	0.69403 (15)	0.15798 (10)	0.0385 (4)	
C22	0.6974 (2)	0.95702 (15)	0.11195 (11)	0.0408 (4)	
C23	0.4379 (2)	0.74181 (17)	0.24467 (11)	0.0435 (5)	
N1	0.3833 (2)	0.81441 (17)	0.18972 (12)	0.0572 (5)	
N2	0.5812 (2)	0.7509 (2)	0.27794 (14)	0.0690 (6)	
O5	0.35758 (16)	0.67037 (14)	0.26518 (9)	0.0594 (4)	
H1N	0.445 (3)	0.857 (2)	0.1710 (13)	0.063 (7)*	
H2N	0.280 (3)	0.807 (2)	0.1663 (16)	0.087 (9)*	
H2	1.189 (3)	0.650 (2)	0.2168 (15)	0.083 (8)*	
H3	0.641 (3)	1.091 (3)	0.1565 (18)	0.100 (10)*	
H3N	0.633 (3)	0.795 (2)	0.2542 (17)	0.083 (9)*	
H4N	0.615 (3)	0.700 (3)	0.3100 (19)	0.093 (11)*	

### Atomic displacement parameters (Å^2^)

**Table:**

	*U*^11^	*U*^22^	*U*^33^	*U*^12^	*U*^13^	*U*^23^
O1	0.0498 (9)	0.0573 (10)	0.0848 (12)	−0.0176 (7)	−0.0043 (8)	0.0312 (9)
O2	0.0416 (8)	0.0458 (8)	0.0690 (10)	−0.0008 (6)	0.0066 (7)	0.0127 (7)
O3	0.1151 (15)	0.0404 (9)	0.0566 (10)	0.0209 (9)	0.0317 (10)	−0.0009 (7)
O4	0.1210 (15)	0.0406 (8)	0.0708 (11)	−0.0034 (9)	0.0585 (11)	0.0006 (8)
C1	0.0404 (10)	0.0274 (9)	0.0344 (9)	−0.0037 (7)	0.0121 (7)	−0.0020 (7)
C2	0.0403 (10)	0.0317 (9)	0.0350 (9)	−0.0020 (7)	0.0120 (7)	−0.0011 (7)
C3	0.0473 (11)	0.0363 (10)	0.0384 (10)	−0.0021 (8)	0.0091 (8)	0.0055 (8)
C4	0.0519 (12)	0.0359 (10)	0.0491 (11)	−0.0097 (9)	0.0153 (9)	0.0079 (8)
C5	0.0490 (12)	0.0401 (11)	0.0712 (14)	−0.0126 (9)	0.0153 (11)	0.0033 (10)
C6	0.0412 (11)	0.0461 (12)	0.0859 (17)	−0.0108 (10)	0.0073 (11)	−0.0033 (12)
C7	0.0447 (11)	0.0411 (11)	0.0705 (14)	−0.0001 (9)	−0.0028 (10)	−0.0010 (10)
C8	0.0447 (11)	0.0336 (10)	0.0539 (12)	−0.0021 (8)	0.0058 (9)	0.0017 (9)
C9	0.0404 (10)	0.0294 (9)	0.0411 (10)	−0.0028 (7)	0.0126 (8)	−0.0027 (7)
C10	0.0424 (10)	0.0326 (9)	0.0484 (11)	−0.0067 (8)	0.0149 (8)	−0.0020 (8)
C11	0.0344 (9)	0.0306 (9)	0.0346 (9)	−0.0048 (7)	0.0069 (7)	0.0010 (7)
C12	0.0387 (10)	0.0311 (9)	0.0395 (10)	−0.0030 (8)	0.0086 (8)	−0.0004 (7)
C13	0.0524 (11)	0.0293 (9)	0.0511 (12)	−0.0001 (8)	0.0134 (9)	0.0049 (8)
C14	0.0566 (12)	0.0386 (11)	0.0460 (11)	−0.0028 (9)	0.0150 (9)	0.0139 (9)
C15	0.0494 (12)	0.0611 (13)	0.0392 (10)	−0.0014 (10)	0.0151 (9)	0.0077 (9)
C16	0.0555 (13)	0.0698 (15)	0.0427 (11)	0.0060 (11)	0.0199 (10)	−0.0055 (11)
C17	0.0571 (13)	0.0484 (12)	0.0536 (12)	0.0049 (10)	0.0181 (10)	−0.0100 (10)
C18	0.0471 (11)	0.0360 (10)	0.0453 (11)	−0.0026 (8)	0.0130 (9)	−0.0021 (8)
C19	0.0328 (9)	0.0343 (9)	0.0346 (9)	−0.0029 (7)	0.0050 (7)	−0.0006 (7)
C20	0.0367 (9)	0.0435 (10)	0.0334 (9)	−0.0024 (8)	0.0061 (7)	0.0043 (8)
C21	0.0421 (10)	0.0381 (10)	0.0362 (9)	−0.0038 (8)	0.0100 (8)	0.0022 (8)
C22	0.0442 (10)	0.0318 (10)	0.0473 (11)	−0.0023 (8)	0.0117 (9)	−0.0026 (8)
C23	0.0412 (10)	0.0495 (12)	0.0415 (10)	−0.0041 (9)	0.0124 (8)	0.0005 (9)
N1	0.0524 (12)	0.0565 (12)	0.0638 (12)	−0.0064 (9)	0.0149 (10)	0.0165 (10)
N2	0.0466 (11)	0.0865 (17)	0.0713 (14)	−0.0114 (11)	0.0071 (10)	0.0190 (13)
O5	0.0469 (8)	0.0691 (10)	0.0601 (9)	−0.0124 (7)	0.0067 (7)	0.0239 (8)

### Geometric parameters (Å, °)

**Table:**

O1—C21	1.203 (2)	C11—C12	1.383 (2)
O2—C21	1.320 (2)	C11—C19	1.428 (2)
O2—H2	1.00 (3)	C12—C13	1.419 (3)
O3—C22	1.312 (2)	C12—C22	1.487 (3)
O3—H3	0.98 (3)	C13—C14	1.352 (3)
O4—C22	1.202 (2)	C13—H13A	0.9300
C1—C2	1.384 (3)	C14—C20	1.407 (3)
C1—C9	1.429 (3)	C14—H14A	0.9300
C1—C11	1.501 (2)	C15—C16	1.354 (3)
C2—C3	1.418 (2)	C15—C20	1.413 (3)
C2—C21	1.488 (3)	C15—H15A	0.9300
C3—C4	1.356 (3)	C16—C17	1.397 (3)
C3—H3A	0.9300	C16—H16A	0.9300
C4—C10	1.406 (3)	C17—C18	1.364 (3)
C4—H4A	0.9300	C17—H17A	0.9300
C5—C6	1.353 (3)	C18—C19	1.414 (3)
C5—C10	1.414 (3)	C18—H18A	0.9300
C5—H5A	0.9300	C19—C20	1.422 (2)
C6—C7	1.401 (3)	C23—O5	1.243 (2)
C6—H6A	0.9300	C23—N1	1.334 (3)
C7—C8	1.361 (3)	C23—N2	1.337 (3)
C7—H7A	0.9300	N1—H1N	0.89 (3)
C8—C9	1.413 (3)	N1—H2N	0.96 (3)
C8—H8A	0.9300	N2—H3N	0.88 (3)
C9—C10	1.423 (3)	N2—H4N	0.85 (3)
			
C21—O2—H2	109.1 (16)	C14—C13—H13A	119.4
C22—O3—H3	113.5 (18)	C12—C13—H13A	119.4
C2—C1—C9	119.32 (16)	C13—C14—C20	120.91 (17)
C2—C1—C11	123.14 (16)	C13—C14—H14A	119.5
C9—C1—C11	117.20 (15)	C20—C14—H14A	119.5
C1—C2—C3	120.09 (16)	C16—C15—C20	121.41 (19)
C1—C2—C21	121.49 (16)	C16—C15—H15A	119.3
C3—C2—C21	118.42 (16)	C20—C15—H15A	119.3
C4—C3—C2	121.10 (18)	C15—C16—C17	120.02 (19)
C4—C3—H3A	119.5	C15—C16—H16A	120.0
C2—C3—H3A	119.5	C17—C16—H16A	120.0
C3—C4—C10	120.76 (17)	C18—C17—C16	120.4 (2)
C3—C4—H4A	119.6	C18—C17—H17A	119.8
C10—C4—H4A	119.6	C16—C17—H17A	119.8
C6—C5—C10	121.1 (2)	C17—C18—C19	121.42 (19)
C6—C5—H5A	119.5	C17—C18—H18A	119.3
C10—C5—H5A	119.5	C19—C18—H18A	119.3
C5—C6—C7	120.3 (2)	C18—C19—C20	117.83 (16)
C5—C6—H6A	119.9	C18—C19—C11	122.32 (16)
C7—C6—H6A	119.9	C20—C19—C11	119.85 (16)
C8—C7—C6	120.4 (2)	C14—C20—C15	122.35 (18)
C8—C7—H7A	119.8	C14—C20—C19	118.80 (17)
C6—C7—H7A	119.8	C15—C20—C19	118.86 (18)
C7—C8—C9	121.25 (19)	O1—C21—O2	122.05 (18)
C7—C8—H8A	119.4	O1—C21—C2	125.36 (17)
C9—C8—H8A	119.4	O2—C21—C2	112.58 (16)
C8—C9—C10	117.99 (17)	O4—C22—O3	121.59 (19)
C8—C9—C1	122.51 (16)	O4—C22—C12	125.48 (17)
C10—C9—C1	119.51 (17)	O3—C22—C12	112.93 (17)
C4—C10—C5	121.86 (18)	O5—C23—N1	121.08 (19)
C4—C10—C9	119.16 (17)	O5—C23—N2	121.6 (2)
C5—C10—C9	118.98 (18)	N1—C23—N2	117.3 (2)
C12—C11—C19	119.17 (16)	C23—N1—H1N	118.9 (16)
C12—C11—C1	123.72 (16)	C23—N1—H2N	116.6 (17)
C19—C11—C1	116.86 (15)	H1N—N1—H2N	123 (2)
C11—C12—C13	119.94 (17)	C23—N2—H3N	115.2 (19)
C11—C12—C22	121.57 (16)	C23—N2—H4N	115 (2)
C13—C12—C22	118.46 (16)	H3N—N2—H4N	127 (3)
C14—C13—C12	121.26 (18)		

### Hydrogen-bond geometry (Å, °)

**Table:**

*D*—H···*A*	*D*—H	H···*A*	*D*···*A*	*D*—H···*A*
N1—H1N···O4	0.89 (3)	2.25 (3)	3.046 (3)	149 (2)
N1—H2N···O1^i^	0.96 (3)	2.10 (3)	3.048 (3)	166 (2)
O2—H2···O5^ii^	1.00 (3)	1.63 (3)	2.606 (2)	162 (2)
O3—H3···O5^iii^	0.98 (3)	1.70 (3)	2.637 (2)	160 (3)
N2—H3N···O4	0.88 (3)	2.17 (3)	3.001 (3)	157 (3)

Symmetry codes: (i) *x*−1, *y*, *z*; (ii) *x*+1, *y*, *z*; (iii) −*x*+1, *y*+1/2, −*z*+1/2.
